# A five-year longitudinal study of the relation between end-stage kidney disease as the outcomes

**DOI:** 10.1186/s12882-020-01795-9

**Published:** 2020-04-15

**Authors:** Hsiu-Lan Li, Pei-Hui Tai, Yi-Ting Hwang, Shih-Wei Lin

**Affiliations:** 1 Graduate Institute of Business and Management, Chang Gung University, Taoyuan City, Taiwan; 2 Department of Nursing, En Cku Kong Hospital, New Taipei City, Taiwan; 3 Department of Statistics, National Taipei University, New Taipei City, Taiwan; 4 Department of Information Management, Chang Gung University, 259 Wen-Hwa 1st Road, Kwei-Shan, Tao-Yuan, 333 Taiwan; 5 Department of Neurology, Linkou Chang Gung Memorial Hospital, Taoyuan City, Taiwan; 6 Department of Industrial Engineering and Management, Ming Chi University of Technology, New Taipei City, Taiwan

**Keywords:** End-stage kidney disease, End-stage renal disease, Longitudinal Study, Comorbidity

## Abstract

**Background:**

Patients with end-stage kidney disease (ESKD) are required to undergo consecutive time-based blood and biochemical tests to determine the progression of the disease according to changes in their blood and biochemical data. This study employed a random intercept model to investigate whether time-based blood and biochemical data present any notable clinical meaning that can be used to track disease progression.

**Methods:**

This study conducted a retrospective analysis on the dialytic data of 148 patients with ESKD, who received hemodialysis between January 2005 and December 2015. The patients were all at least 20 years old, and the data used included patient demographic information and results for at least 60 blood and biochemical tests. A random intercept model was used to analyze the relationships among blood and biochemical test results, explanatory variables of patient comorbidities, and time.

**Results:**

The age range of patients was between 33 and 98 years, with an average of 66.1 years and those over 65 years old comprising 51.3% (*n* = 76) of the total. Furthermore, hypertension was found to be the most common comorbidity among patients (87.2%, *n* = 129), followed by anemia (48.6%, *n* = 72), diabetes (47.3%, *n* = 70), dyslipidemia (19.6%, *n* = 29), and peptic ulcer (19.6%, *n* = 29). Coronary atherosclerotic heart disease is a comorbidity that can serve as a strong and independent marker for prognosis in patients with ESKD. Serum creatinine level can serve as an alternative indicator because patients with ESKD and comorbid diabetes may exhibit increased creatinine levels.

**Conclusions:**

The results of a parameter estimation for longitudinal data analysis suggested that comorbidity and time were critical variables influencing blood and biochemical test results. Furthermore, WBC and HBC, HCT, albumin, protein, and creatinine levels were recognized as variables of critical significance. The results obtained in this study indicate that multimorbidity increases the treatment burden on patients, leading to polypharmacy. For this reason, comprehensive care and treatment of ESKD cannot rely solely on data from one single time point; instead, longitudinal analysis and other data that can affect patient prognosis must also be considered.

## Background

Hemodialysis is a type of renal replacement therapy that is particularly effective for removing uremic toxins, rectifying body fluid abnormalities, and achieving electrolyte and (temporary) pH balance [1, 2]. However, monitoring renal functions and various biochemical data is essential in hemodialysis for ESKD because it helps to assess disease progression [3]. Normally, patient blood and biochemical data are repeatedly obtained after the patient has undergone hemodialysis to monitor for signs of kidney failure or associated comorbidities. These data include platelet count, white blood cell count (WBC), and alanine transaminase (ALT), aspartate transaminase (AST), albumin, alkaline, protein, total bilirubin, hemoglobin count (HBC), hematocrit (HCT), mean corpuscular volume (MCV), creatinine, K, Na, uric acid, Ca, and P levels [4–6]. These blood and biochemical test items are all related to kidney functions, and each has its own limitations and can be affected by patient demographic or physiological characteristics. Therefore, they must all be measured to ensure accurate evaluation [7, 8]. In Taiwan, multi-morbidities and their prevalence rates among patients receiving hemodialysis in 2015 were discovered to be as follows: hypertension (82%), cardiovascular diseases (56.2%), diabetes (49.3%), dyslipidemia (32.7%), and peptic ulcer (29.6%) [4]. This indicates that in most medical or research settings, deteriorating conditions must not be diagnosed according to a single symptom or sign; rather, multiple items must be investigated to ensure an accurate diagnosis [9, 10].

Patients with ESKD are required to undergo consecutive time-based blood and biochemical tests to determine the progression of a disease [11]. In several studies on comorbidities associated with ESKD, researchers have reported that patients with ESKD who receive renal replacement therapy usually develop one or multiple comorbidities or complications [12, 13]. Studies have predominantly involved obtaining risk factors from baseline. They lacked longitudinal data of biomarkers (e.g. Ca-P) to derive risk factors, and few have considered changes in patient conditions at different time points [8, 14]. The present study employed a random intercept model, which is widely used and has the advantage of enabling the application of longitudinal analysis by repeatedly observing changes in an individual over time. This model is ideal for experiments that are particularly short or long [15–17]. This study employed a random intercept model to investigate whether time-based blood and biochemical data present any notable clinical meaning that can be used to track disease progression.

## Methods

### Ethics statement

This study was approved by the Institutional Review Board Committee, En Cku Kong Hospital (ECKIRB1060801), and the experimental practices adopted conformed entirely to the Declaration of Helsinki. The low-risk nature of this study warranted the use of retrospective methods for the analysis of data that were readily available, thus nullifying the necessity of obtaining informed consent from patients. The data owner also granted permission for the use of all relevant data for research purposes in this study.

### Data collection

**Fig. 1:**
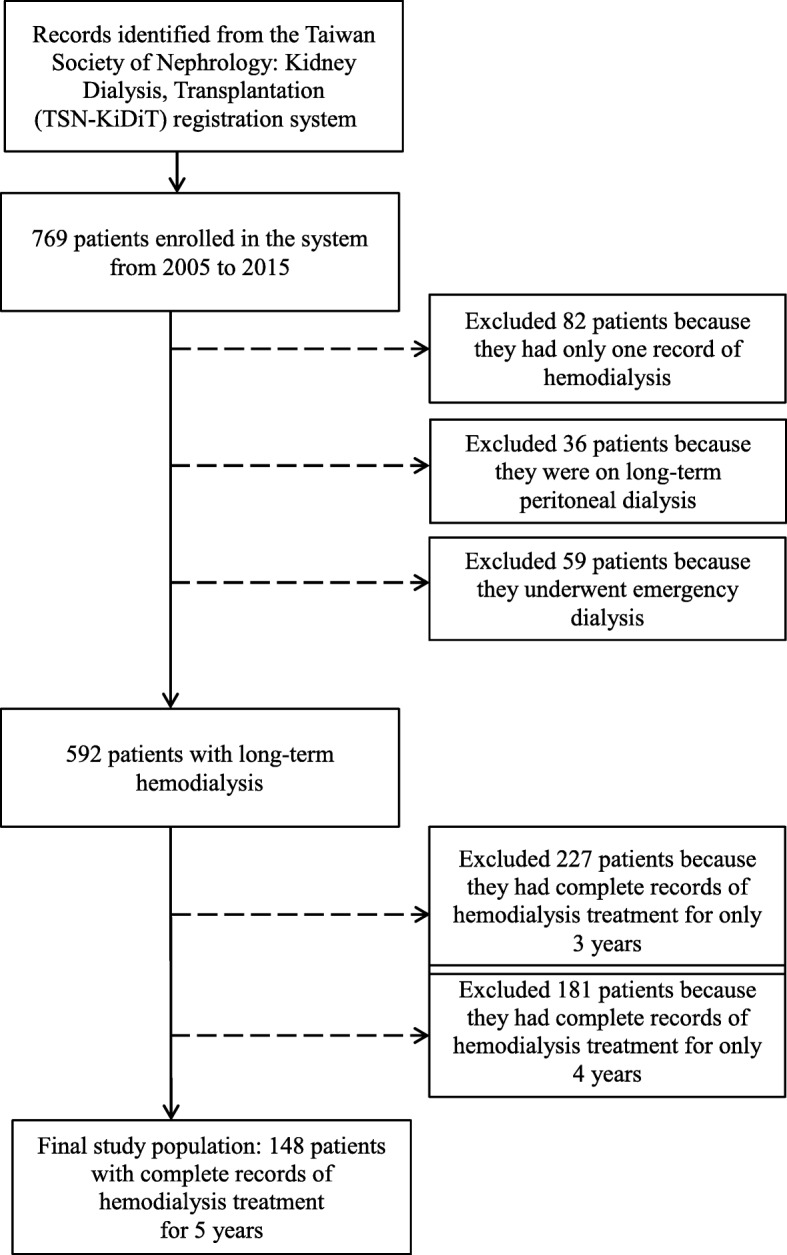
Patient flow chart of this study

Data used in this study were obtained from the Taiwan Society of Nephrology: Kidney Dialysis, Transplantation (TSN-KiDiT) registration system. These data included information on the survival rate, cause, treatment modality, and biochemical laboratory results of end-stage kidney disease (ESKD). Data of 769 patients from 2005 to 2015 were available in the system. We excluded 177 of the 769 patients who had only one recorded hemodialysis treatment. Among 592 eligible patients, only 148 patients had complete records of hemodialysis treatment for 5 years (Fig. 1). The sample size was not pre-determined. The number of eligible patients were completely determined from retrospectively reviewing all the medical records in 2005–2015 for hemodialysis from one hospital, ESKD. We wanted to study the pattern of change for the patient blood and biochemical data. The final eligible subjects were 148. Since it was an observational study, to reduce the bias, age, gender, duration of hemodialysis and comorbidities were included when obtaining the estimates for the model. The study site was a teaching hospital in Taiwan. Patients were included in the study if they were at least 20 years of age and were undergoing hemodialysis for ESKD [18]. Data from a medical teaching hospital from January 2005 to December 2015 were used in this study, which comprised 5-year longitudinal data of at least 60 blood and biochemical tests results of patients. In this retrospective study, the dialysis data of 148 patients with ESKD, comprising the treatment codes and the International Classification of Diseases Ninth Revision Clinical Modification (ICD-9-CM) codes, were analyzed. Patients who underwent kidney transplantation and who received peritoneal dialysis treatment were excluded. All of the included patients had been diagnosed with ESKD by nephrologists on the basis of the 2012 Kidney Disease: Improving Global Outcomes (KDIGO) guidelines, wherein disease severity was determined based on the cause, glomerular filtration rate (GFR), and albuminuria categories (CGA) [19]. Patients with ESKD, the fifth stage of chronic kidney disease (CKD), have a GFR value of < 15 mL/min/1.73 m^2^ [18]. In this study, for patients with ESKD, the demographic data (including age and sex) and test results of at least 60 blood and biochemical tests (including duration of the hemodialysis treatment, comorbidities associated with ESKD, and relevant dialysis data) were used. This study used 2006–2011 data on registered patients in Taiwan who had ESKD and were undergoing dialysis. The mean age at registration was 64.34 ± 14.2 years [20]. Those aged > 65 years were considered older adults [4]. Subjects more than 65 years of age were considered elderly. To analyze ESKD-associated comorbidities, patients were categorized according to each comorbidity [4, 11, 21, 22]. A total of 14 comorbidities (ICD-9-CM) were noted, which were as follows: diabetes, cerebrovascular accident, heart failure, heart arrhythmia, coronary atherosclerotic heart disease (CAHD), anemia, dyslipidemia, chronic obstructive pulmonary disease (COPD), peptic ulcer, atrial fibrillation, hypertension, cardiovascular diseases, ischemic heart disease (IHD), and ischemic stroke. Patients regularly attended hemodialysis sessions and underwent blood and biochemical tests at the same hospital once every month throughout the duration of the hemodialysis treatment. On the basis of the dialysis quality indices of the inspection and review standards for hemodialysis and peritoneal dialysis specified by the Taiwan Society of Nephrology, as well as the clinical performance indicators revealed in the Taiwan Renal Registry Data System [4, 11], the following blood and biochemical test indicators were included in the study: platelet and WBC counts and ALT, AST, albumin, alkaline, protein, total bilirubin, HBC, HCT, MCV, creatinine, K, Na, uric acid, Ca, and P levels.

### Statistical analysis

The time plot of blood and biochemical test versus time in months, with Lowess smoothed curve, was used to investigate the potential time trend of blood and biochemical tests. The random intercept model with a linear trend was then used to assess the long-term association between blood and biochemical tests and comorbidities. All of the interactions between time and comorbidities were also included in the model and were excluded when the corresponding *p* value was smaller than 0.05. Data processing and statistical analyses were conducted using SAS version 9.4 for Windows (SAS institute Inc., Cary, NC, USA).

## Results

In this retrospective study, all of the included patients were enrolled in the TSN-KiDiT registration system. Data of 769 patients were available in the system for the period of 2005 to 2015. Of the 769 patients, we excluded 177 who had records of only one hemodialysis treatment or who received a long-term peritoneal dialysis or emergency dialysis treatment. Among the 592 eligible patients, only 148 had complete records of hemodialysis treatment for 5 years. Thus, the final study population comprised 148 patients (Fig. 1). Table 1 presents patient demographic data, which reveals that more women (54%) than men were included in the study and that the mean patient age was 66.1 years (range: 33–98 years), with 51.3% patients more than 65 years old (*n* = 76). Patients were categorized according to hemodialysis treatment duration into beginner (*n* = 57, 38.51%), less than 1 year (*n* = 38, 25.68%), and more than 1 year (*n* = 53, 35.81%) groups. Moreover, most of the patients had one or more of the following comorbidities: diabetes (*n* = 70, 47.3%), hypertension (*n* = 129, 87.2%), dyslipidemia (*n* = 29, 19.6%), cardiovascular disease (*n* = 14, 9.5%), IHD (*n* = 18, 12.2%), heart failure (*n* = 51, 34.5%), CAHD (*n* = 61, 41.2%), anemia (*n* = 72, 48.7%), heart arrhythmia (*n* = 30, 20.3%), atrial fibrillation (*n* = 6, 4.1%), cerebrovascular accident (*n* = 20, 13.5%), ischemic stroke (*n* = 8, 5.4%), COPD (*n* = 6, 4.1%), and peptic ulcer (*n* = 29, 19.6%).

**Table 1:** Patient demographic characteristics

Variable	Categories	No. of patients	Percent
Gender	Female	80	54.05
	Male	68	45.95
Age	< 65	72	48.65
	> = 65	76	51.35
Duration of hemodialysis treatment	Beginer	57	38.51
	Less than 1 year	38	25.68
	More than 1 years	53	35.81
Comorbidities			
Diabetes	No	78	52.70
	Yes	70	47.30
Hypertension	No	19	12.84
	Yes	129	87.16
Dyslipidemia	No	119	80.41
	Yes	29	19.59
Cardiovascular disease	No	134	90.54
	Yes	14	9.46
Ischemic heart disease	No	130	87.84
	Yes	18	12.16
Heart failure	No	97	65.54
	Yes	51	34.46
Coronary atherosclerotic heart disease	No	87	58.78
	Yes	61	41.22
Anemia	No	76	51.35
	Yes	72	48.65
Heart arrhythmia	No	118	79.73
	Yes	30	20.27
Atrial fibrillation	No	142	95.95
	Yes	6	4.05
Cerebrovascular accident	No	128	86.49
	Yes	20	13.51
Ischemic stroke	No	140	94.59
	Yes	8	5.41
Chronic obstructive pulmonarydisease	No	142	95.95
	Yes	6	4.05
Peptic ulcer	No	119	80.41
	Yes	29	19.59

Table 2 Subgroup analysis especially for gender as sensitivity analysis for the outcomes. The top three comorbid diseases in female patients were hypertension (*n* = 66, 82.5%), anemia (*n* = 38, 47.5%), and diabetes (*n* = 32, 40.0%). The top three comorbid diseases in male patients were hypertension (*n* = 63, 92.6%), diabetes (*n* = 38, 55.8%), and anemia (*n* = 34, 50.0%).

**Table 2:** Subgroup analysis especially for gender as sensitivity analysis for the outcomes

Variable	Category	Female		Male	
		n	%	n	%
Diabetes	No	48	60.00	30	44.12
	Yes	32	40.00	38	55.88
Hypertension	No	14	17.50	5	7.35
	Yes	66	82.50	63	92.65
Dyslipidemia	No	65	81.25	54	79.41
	Yes	15	18.75	14	20.59
Cardiovascular disease	No	71	88.75	63	92.65
	Yes	9	11.25	5	7.35
Ischemic heart disease	No	71	88.75	59	86.76
	Yes	9	11.25	9	13.24
Heart failure	No	50	62.50	47	69.12
	Yes	30	37.50	21	30.88
Coronary atherosclerotic heart disease	No	50	62.50	37	54.41
	Yes	30	37.50	31	45.59
Anemia	No	42	52.50	34	50.00
	Yes	38	47.50	34	50.00
Heart arrhythmia	No	64	80.00	54	79.41
	Yes	16	20.00	14	20.59
Atrial fibrillation	No	76	95.00	66	97.06
	Yes	4	5.00	2	2.94
Cerebrovascular accident	No	73	91.25	55	80.88
	Yes	7	8.75	13	19.12
Ischemic stroke	No	77	96.25	63	92.65
	Yes	3	3.75	5	7.35
Chronic obstructive pulmonary disease	No	77	96.25	65	95.59
	Yes	3	3.75	3	4.41
Peptic ulcer	No	61	76.25	58	85.29
	Yes	19	23.75	10	14.71

Table 3 Subgroup analysis especially for age as sensitivity analysis for the outcomes. The top three comorbid diseases in patients aged < 65 years were hypertension (*n* = 53, 79.1%), anemia (*n* = 27, 40.3%), and diabetes (*n* = 26, 38.8%). The top three comorbid diseases in patients aged ≥65 years were hypertension (*n* = 76, 93.8%), anemia (*n* = 45, 55.5%), and diabetes (*n* = 44, 54.3%).

**Table 3:** Subgroup analysis especially for age as sensitivity analysis for the outcomes

Variable	Category	< 65		> = 65	
		n	%	n	%
Diabetes	No	41	61.19	37	45.68
	Yes	26	38.81	44	54.32
Hypertension	No	14	20.90	5	6.17
	Yes	53	79.10	76	93.83
Dyslipidemia	No	55	82.09	64	79.01
	Yes	12	17.91	17	20.99
Cardiovascular disease	No	62	92.54	72	88.89
	Yes	5	7.46	9	11.11
Ischemic heart disease	No	60	89.55	70	86.42
	Yes	7	10.45	11	13.58
Heart failure	No	55	82.09	42	51.85
	Yes	12	17.91	39	48.15
Coronary atherosclerotic heart disease	No	48	71.64	39	48.15
	Yes	19	28.36	42	51.85
Anemia	No	40	59.70	36	44.44
	Yes	27	40.30	45	55.56
Heart arrhythmia	No	61	91.04	57	70.37
	Yes	6	8.96	24	29.63
Atrial fibrillation	No	66	98.51	76	93.83
	Yes	1	1.49	5	6.17
Cerebrovascular accident	No	63	94.03	65	80.25
	Yes	4	5.97	16	19.75
Ischemic stroke	No	64	95.52	76	93.83
	Yes	3	4.48	5	6.17
Chronic obstructive pulmonary disease	No	65	97.01	77	95.06
	Yes	2	2.99	4	4.94
Peptic ulcer	No	56	83.58	63	77.78
	Yes	11	16.42	18	22.22

Additional file 1: Appendix 1 displayed the parameter estimates of the random intercept model for HBC, HCT, MCV, platelet, and WBC. The estimated variance of the random intercept was significant, which implied some heterogeneity among the study samples. The findings were as follows:

### HBC

The interactions between time and 7 conditions, namely diabetes, cerebrovascular accident, heart failure, atrial fibrillation, hypertension, ischemic heart disease, and ischemic stroke were significantly associated with HBC. Compared with the reference group, the change over time of HBC for patients with diabetes, cerebrovascular accident, heart failure, and atrial fibrillation was negative and significant. Male patients had a significantly higher HBC count than female patients did. Patients who had anemia had a significantly lower HBC. For every record of increase in inpatient times, the average HBC declined by 0.021 units.

### HCT

The interactions between time and 7 conditions, namely cerebrovascular accident, heart failure, heart arrhythmia, atrial fibrillation, hypertension, ischemic heart disease, and ischemic stroke were significantly associated with HCT. The slope for patients without any diseases was nonsignificant: The average HCT remained stable over time. Compared with the reference group, the HCT change over time for patients with cerebrovascular accident, heart failure, heart arrhythmia, and atrial fibrillation was negative and significant. Male patients had a significantly higher HCT than female patients did. Patients who had anemia had significantly lower HCT.

### MCV

The interactions between time and 8 conditions, namely diabetes, cerebrovascular accident, heart failure, heart arrhythmia, anemia, chronic obstructive pulmonary disease, peptic ulcer, and cardiovascular disease were significantly associated with MCV. For every 1-year increase in age, the average MCV increased significantly by 0.131 units. Patients who had hypertension had significantly lower MCV. For every record of increase in inpatient times, the average MCV declined by 0.140 units.

### Platelet count

The interactions between time and 6 conditions, namely diabetes, cerebrovascular accident, heart arrhythmia, anemia, chronic obstructive pulmonary disease, and cardiovascular disease were significantly associated with platelet count. The change over time of platelet count for patients with cardiovascular disease, chronic obstructive pulmonary disease, and cardiovascular disease was significant and positive compared with the reference group. For every 1-year increase in age, the average platelet count dropped by1.014 units.

### WBC

The interactions between time and 10 conditions, namely diabetes, cerebrovascular accident, heart arrhythmia, anemia, chronic obstructive pulmonary disease, peptic ulcer, atrial fibrillation, hypertension, cardiovascular disease, and ischemic stroke were significantly associated with WBC. The slope for patients without any diseases was nonsignificant. The average WBC remained stable as time increased. Compared with the reference group, the change over time of WBC for patients with diabetes, hypertension, and ischemic stroke was negative and significant. Patients who had diabetes also had significantly higher WBC, whereas patients who had heart arrhythmia had significantly lower WBC. For every record of increase in inpatient times, the average WBC increased by 0.046 units.

Additional file 1: Appendix 2 displays the parameter estimates of the random intercept model for ALT, AST, albumin, alkaline, protein, and total bilrubin. The estimated variance of the random intercept was significant, which implied that some heterogeneity existed in the study sample. The findings were as follows:

### ALT

The interactions between time and 6 conditions, namely cerebrovascular accident, heart failure, anemia, peptic ulcer, cardiovascular disease, and ischemic stroke were significantly associated with ALT. Male patients had significantly higher ALT than female patients did. Patients who had cardiovascular disease had significantly higher ALT than those without did.

### AST

Interactions between time and cardiovascular disease were significantly associated with AST. Patients with peptic ulcer had significantly higher AST.

### Albumin

The interactions between time and 9 conditions, namely diabetes, cerebrovascular accident, heart arrhythmia, coronary atherosclerotic heart disease, hypertension, cardiovascular disease, and ischemic heart disease were significantly associated with albumin. Patients with chronic obstructive pulmonary disease had significantly lower albumin.

### Alkaline

The interactions between time and 9 conditions, namely cerebrovascular accident, heart failure, dyslipidemia, chronic obstructive pulmonary disease, peptic ulcer, hypertension, cardiovascular disease, ischemic heart disease, and ischemic stroke were significantly associated with alkaline levels. Compared with the reference group, the change over time of albumin for patients with cerebrovascular accident, heart failure, chronic obstructive pulmonary disease, hypertension, ischemic heart disease, and ischemic stroke was significantly positive. Patients who had peptic ulcer had significantly higher alkaline levels. For every record of increase in inpatient times, the average alkaline increased by 1.087 units.

### Protein

The interactions between time and 11 conditions, namely diabetes, heart failure, heart arrhythmia, anemia, dyslipidemia, chronic obstructive pulmonary disease, peptic ulcer, hypertension, cardiovascular disease, ischemic heart disease, and ischemic stroke were significantly associated with protein levels. Compared with the reference group, the change over time in protein for patients with diabetes, heart failure, anemia, chronic obstructive pulmonary disease, and hypertension, were significantly positive. Patients who had chronic obstructive pulmonary disease had significantly higher protein levels.

### Total bilrubin

Interaction between time and cardiovascular disease was significantly associated with total bilrubin. Patients who had anemia demonstrated significantly lower total bilrubin.

Additional file 1: Appendix 3 displays the parameter estimates of the random intercept model for creatinine, K, Na, uric acid, and Ca. The estimated variance of the random intercept was significant, which implied that some heterogeneity existed in the study sample. The findings were as follows:

### Creatinine

The interactions between time and 8 conditions, namely diabetes, heart failure, heart arrhythmia, coronary atherosclerotic heart disease, dyslipidemia, peptic ulcer, atrial fibrillation, and ischemic stroke were significantly associated with creatinine. Male patients had significantly higher creatinine. For every 1-year increase in age, the average creatinine level dropped significantly, by 0.065 units. For every record of increase in inpatient times, the average creatinine level decreased significantly, by 0.045 units.

### K

The interactions between time and 7 diseases, namely heart arrhythmia, coronary atherosclerotic heart disease, dyslipidemia, chronic obstructive pulmonary disease, cardiovascular disease, ischemic heart disease, and ischemic stroke were significantly associated with K concentration. Patients who had diabetes demonstrated significantly lower K levels.

### Na

The interactions between time and 5 conditions, namely diabetes, anemia, peptic ulcer, atrial fibrillation, and hypertension were significantly associated with Na levles. Patients who had diabetes had significantly lower Na levels.

### Uric acid

The interactions between time and 4 conditions, namely heart arrhythmia, hypertension, cardiovascular disease, and ischemic stroke were significantly associated with uric acid levels. For every 1-year increase in age, patients had significantly lower uric acid levels. Patients who had had ischemic stroke had significantly higher uric acid levels.

### Ca

The interactions between time and 5 conditions, namely heart arrhythmia, dyslipidemia, chronic obstructive pulmonary disease, hypertension, and ischemic heart disease were significantly associated with Ca. For every record of increase in inpatient times, the average Ca concentration decreased by 0.045 units, which was significant.

## Discussion

The study population comprised 148 patients, namely 80 women and 68 men, with an approximately equal number of patients in each age group. This study discovered that in terms of sex, hypertension was the most prevalent comorbid disease in both male and female patients. This finding concurs with that of previous reports, which stated that hypertension was a prevalent disease in patients receiving dialysis regularly [19, 23]. In terms of age, the population of older adults and increasing prevalence of the causative comorbiditie has increased rap-idly. In this epidemiologic data, 51.3% (*n* = 76) of the patients with ESKD were ≥ 65 years old. That is, patients ≥65 years old had a higher incidence of ESKD than patients < 65 years old. In the recent decades, The prevalence rate of ESRD patients in older adults is higher than that in adolescents [24–26]. The data presented in three related studies also indicated that the incidence of ESKD increased with age [27–29]. The present study identified hypertension (87.2%, *n* = 129) as the most common comorbidity, followed by anemia (48.6%, *n* = 72), diabetes (47.3%, *n* = 70), dyslipidemia (19.6%, *n* = 29), and peptic ulcer (19.6%, *n* = 29). The discovery that hypertension and diabetes were among the most common comorbidities was in agreement with several related studies [4, 13, 30]. Notably, this study discovered that anemia was the second most common comorbidity, which is in agreement with the findings of another study [31]. The prevalence of anemia as a comorbidity among patients receiving hemodialysis can be attributed to the increased risk of anemia following the development of ESKD [32].

In this study, we investigated whether the influence of patient comorbidities as explanatory variables changed with time. Demographic data from the TSN-KiDiT system were included as categorical variables in a random intercept model for longitudinal analysis. The parameter estimation results of the longitudinal data set indicated that comorbidity and time were critical variables that influenced the blood and biochemical test results. Furthermore, WBC counts and HBC, HCT, albumin, total protein, and creatinine levels were found to be variables of critical significance. Because studies have suggested that most patients with ESKD can develop one or multiple comorbidities [12, 13], the 3 most common comorbidities, namely hypertension (87.2%), anemia (48.6%), and diabetes (47.3%), were analyzed in this study. The other comorbidities that were studied were dyslipidemia and peptic ulcer. Furthermore, multimorbidity may increase the treatment burden on patients, leading to polypharmacy. Therefore, administering treatment for these comorbidities was necessary for preventing them from inducing or expediting the occurrence of inflammation [33].

One study reported that comorbidities can be the reason for poor prognosis in patients with ESKD or serve as a marker for poor prognosis in patients receiving dialysis and that a low serum albumin concentration may indicate malnutrition [34]. A clinical epidemiological study further suggested that arterial damage is the major cause of high cardiovascular incidence and mortality rates among patients with ESKD [35]. Although the present study was unable to provide direct evidence, coronary atherosclerotic heart disease was noted as a comorbidity that could be a strong and independent marker for prognosis of patients with ESKD.

In estimating parameters for the longitudinal analysis, this study discovered that the estimation results could be used to explain the time-dependent changes of variables in the random intercept model. Among the common comorbidities, diabetes was observed to correspond to decreasing albumin levels over time, with a trend of − 0.001 (− 0.001 to 0.000). This indicates that in the long term, patients with ESKD who have also developed diabetes may exhibit decreasing albumin levels because of the comorbidity [36]. Alternatively, serum creatinine level can serve as an indicator because patients with ESKD who have also developed diabetes can exhibit increased creatinine levels [37].

To explore the trend of biological indicators exhibited by patients who received hemodialysis in the past 5 years, this study delved into the interactions between the two variables—time and comorbidity—in the proposed model. The parameter estimates of the random intercept model for blood and biological indicators were applicable to verifying the effect of comorbidity on the outcome of patients with ESKD. Results obtained in this study indicate that the interaction between time and cerebrovascular accidents is significantly correlated with blood; the interaction between time and diabetes is significantly correlated with HBC, MCV, PLT, WBC, Creatinine, and Na levels; the interaction between time and hypertension is significantly correlated with HBC, HCT, WBC, uric acid, Na, Ca levels. The trend of these biological indicators exhibited by patients who have received hemodialysis may enable medical staff to evaluate comorbidities and address progressive disease imposing high risks. The results also suggest an acute need to establish customized ESKD nursing strategies for high-risk populations, in addition to the existing 2012 Kidney Disease: Improving Global Outcomes guidelines, which are abided by medical staff to treat chronic kidney disease.

Health education regarding chronic kidney disease should be provided for patients with ESKD. ESKD care aims to improve patients’ quality of life and delay kidney function deterioration, which can lead to end-stage kidney failure (dialysis) and complications. The objective and priority of ESKD care depend on patients’ understanding of kidney disease. Therefore, such care should be implemented from the following perspectives: (1) promotion of effective physician–patient communication, (2) introduction of adequate concepts regarding treatment seeking and regular follow-ups, (3) development of correct eating habits and lifestyle, (4) early kidney health education and interventions for controlling blood pressure, blood lipid, blood sugar, and nutrient levels and treating comorbid diseases such as anemia, and (5) enhancement of self-care ability [38, 39].

This was a longitudinal and retrospective study. Patients must be followed-up for at least 5 years and have 60 dialysis outcomes to be included. Each outcome was measured 60 times through hematological or biochemical tests to determine the duration of hemodialysis treatment, detect comorbidities associated with ESKD, and obtain other relevant dialysis data; 16 outcomes (platelet and WBC counts as well as levels of ALT, AST, albumin, alkaline, protein, total bilirubin, HBC, HCT, MCV, creatinine, K, Na, uric acid, Ca, and P) were considered. First, it had access to the comorbidity data of at least 60 blood and biochemical tests, and these data were of high quality because they were provided by a large kidney registration system [40]. Second, this study was able to apply annual data sets on the time-based changes in the explanatory variables of 14 comorbidities, making this study the first to do so worldwide.

The results of this study showed that correlations existed between ESKD-related comorbidity, time, blood data, and biochemical data. This study comprehensively collected conventional risk factors and comorbidities related to ESKD. A longitudinal study was conducted to track the patients, approximately 30 covariates were included in the model, and it was conducive to precisely record the patient’s kidney functions and prognosis. This study indicated the importance of controlling comorbidities related to ESKD. The results of this study influence clinical and research practices. Through combining the testing data of patients undergoing dialysis, we made those patients’ information more comprehensive. We made a breakthrough in the topics of studying the biochemical values of patients undergoing dialysis. The values brought by this study would be conducive to analyzing epidemiology and related risk factors among patients undergoing dialysis in Taiwan, achieving the goals of preventing kidney diseases, increasing the survival rate of patients undergoing dialysis, and reducing related comorbidities.

This study had certain limitations. For example, the data used in this study were obtained from the TSN-KiDiT registration system. The software TSN-KiDiT lacks indications of critical clinical performance under that subject. For example, it lacks the self-aware health information, quality of life, living environment, exercise habit, smoking history, medication, and family member’s disease history of patients with ESKD. These factors may aggravate the disease. Second, regarding patients who had been transferred to another hospital, their TWRDS data were concentrated in the drop-outs state, and this study was not authorized to read patients’ data after they had been transferred to another hospital. Furthermore, several other comorbidities have been reported to be associated with ESKD, such as cancer [41] and thyroid disease [42]. The lack of disease data in the present study setting might have caused result underestimation, leading to the conclusion that a certain disease is not associated with ESKD. Furthermore, other limitations of this retrospective longitudinal study include population size, single hospital, extensive time span (10-year data), and potential differences in effects at various time points, which were the primary factors hindering data interpretation. Inclusion criteria of this study were that patients must be followed for at least 5 years and have 60 dialysis outcomes. Thus, patients without 5-year data were excluded, which is another limitation of this study. However, most methods can currently only address the influence of an individual comorbidity and its related factors at a specific time point. The study population comprised 148 patients, namely 80 women and 68 men, with an approximately equal number of patients in each age group. This study considered 14 chronic diseases, the hemodialysis treatment duration, and the number of recorded inpatient treatments. The controlling variables were analyzed using a linear mixed model, which included the possible interaction between the time and chronic diseases. This study included approximately 30 covariates in the model. In addition, other possible limitations of this study are that the prevalence of some of the chronic diseases in the study population might have been extremely high or low (i.e., few patients in one group; supplementary data) and that the subgroup analysis might not have been robust. This study used only data collected from a hospital. The sample size was determined by all eligible patients in this hospital. Some variability might not be taken into according. This becomes the study limitation. Future prospective studies involving larger patient cohorts are, therefore, needed to confirm this study findings.

## Conclusions

This study investigated whether the explanatory variables of comorbidities associated with ESKD change over time. The results of a parameter estimation for longitudinal data analysis suggested that comorbidity and time were critical variables influencing blood and biochemical test results. Furthermore, WBC and HBC, HCT, albumin, protein, and creatinine levels were recognized as variables of critical significance. The results obtained in this study indicate that multimorbidity increases the treatment burden on patients, leading to polypharmacy. For this reason, comprehensive care and treatment of ESKD cannot rely solely on data from one single time point; instead, longitudinal analysis and other data that can affect patient prognosis must also be considered.

## Supplementary information

**Additional file 1.** :Appendix 1. Estimation results of time-based explanatory variables in the random intercept model for a blood test (HBC, HCT, MCV, platelet, WBC). Appendix 2. Estimation results of time-based explanatory variables in the random intercept model for a biochemical test (ALT, AST, albumin, alkaline, protein, total bilirubin levels). Appendix 3. Estimation results of time-based explanatory variables in the random intercept model for a biochemical test(Creatinine, K, Na, Uric acid, Ca).

## Data Availability

This study can confirm this study have included a statement regarding data and material availability in the declaration section of my manuscript. The datasets used and/or analysed during the current study are available from the corresponding author on reasonable request.

## Abbreviations

ESKD: End-stage kidney disease
WBC: White blood cell count
ALT: Alanine transaminase
AST: Aspartate transaminase
HBC: Hemoglobin count
HCT: Hematocrit
MCV: Mean corpuscular volume
TSN-KiDiT: Taiwan Society of Nephrology: Kidney Dialysis, Transplantation
ICD-9-CM: International Classification of Diseases Ninth Revision Clinical Modification
KDIGO: Kidney Disease: Improving Global Outcomes
GFR: Glomerular filtration rate
CGA: Albuminuria categories
CAHD: Coronary atherosclerotic heart disease
COPD: Chronic obstructive pulmonary disease
CD: Cardiovascular diseases
IHD: Ischemic heart disease
