# Supplementary material for: A five-year longitudinal study of the relation between end-stage kidney disease as the outcomes
Source: BMC Nephrol. 2020 Apr 15;21:132. doi: 10.1186/s12882-020-01795-9 (PMC7161172; doi:10.1186/s12882-020-01795-9)
Supplement: Supplementary file 1 — Additional file 1. :Appendix 1. Estimation results of time-based explanatory variables in the random intercept model for a blood test (HBC, HCT, MCV, platelet, WBC). Appendix 2. Estimation results of time-based explanatory variables in the random intercept model for a biochemical test (ALT, AST, albumin, alkaline, protein, total bilirubin levels). Appendix 3. Estimation results of time-based explanatory variables in the random intercept model for a biochemical test(Creatinine, K, Na, Uric acid, Ca). [file 12882_2020_1795_MOESM1_ESM.docx]

Appendix 1. Estimation results of time-based explanatory variables in the random intercept model for a blood test (HBC, HCT, MCV, platelet, WBC)

| Variable Categorical | | Outcomes | | | | | | | | | | | | | | |
| --- | --- | --- | --- | --- | --- | --- | --- | --- | --- | --- | --- | --- | --- | --- | --- | --- |
|  |  | HBC | | | HCT | | | MCV | | | Platelet | | | WBC | | |
|  |  | Estimate | SE | P | Estimate | SE | P | Estimate | SE | P | Estimate | SE | P | Estimate | SE | P |
| Intercept |  | 9.810 | 0.386 | <.001* | 29.036 | 1.163 | <.001* | 88.231 | 3.453 | <.001* | 243.25 | 23.002 | <.001* | 7.052 | 0.733 | <.001* |
| Male |  | 0.809 | 0.148 | <.001* | 2.067 | 0.445 | <.001* | 1.999 | 1.332 | 0.133 | -9.445 | 8.836 | 0.285 | 0.108 | 0.281 | 0.701 |
| Age |  | 0.007 | 0.006 | 0.205 | 0.024 | 0.017 | 0.168 | 0.131 | 0.051 | 0.011* | -1.014 | 0.340 | 0.003* | -0.013 | 0.011 | 0.235 |
| Duration of hemodialysis treatment | Less than 1 year | -0.249 | 0.179 | 0.164 | -0.505 | 0.540 | 0.350 | -0.290 | 1.611 | 0.857 | -0.634 | 10.728 | 0.953 | -0.000 | 0.341 | 1.000 |
|  | More than 1years | -0.535 | 0.170 | 0.002* | -1.391 | 0.512 | 0.007* | -0.603 | 1.519 | 0.691 | 21.327 | 10.165 | 0.036* | -0.638 | 0.323 | 0.048* |
| Diabetes |  | 0.146 | 0.171 | 0.394 | 0.160 | 0.503 | 0.750 | -1.698 | 1.517 | 0.263 | 16.234 | 10.127 | 0.109 | 0.740 | 0.325 | 0.023* |
| Cerebrovascular accident |  | 0.078 | 0.223 | 0.728 | 0.595 | 0.671 | 0.375 | -2.040 | 1.950 | 0.295 | 0.843 | 13.162 | 0.949 | 0.520 | 0.422 | 0.218 |
| Heart failure |  | -0.219 | 0.173 | 0.205 | -0.542 | 0.522 | 0.299 | -2.256 | 1.515 | 0.137 | 2.083 | 10.106 | 0.837 | 0.190 | 0.321 | 0.553 |
| Heart arrhythmia |  | 0.367 | 0.224 | 0.101 | 1.289 | 0.690 | 0.062 | -0.175 | 2.008 | 0.930 | 8.012 | 13.553 | 0.554 | -0.679 | 0.435 | 0.119 |
| CAHD |  | 0.019 | 0.184 | 0.920 | -0.080 | 0.555 | 0.886 | 2.287 | 1.649 | 0.166 | -0.024 | 11.024 | 0.998 | 0.161 | 0.358 | 0.653 |
| Anemia |  | -0.496 | 0.156 | 0.001* | -1.346 | 0.470 | 0.004* | -1.744 | 1.407 | 0.215 | 19.755 | 9.446 | 0.037* | 0.287 | 0.303 | 0.344 |
| Dyslipidemia |  | 0.304 | 0.194 | 0.117 | 0.928 | 0.585 | 0.112 | 1.354 | 1.737 | 0.436 | 5.654 | 11.769 | 0.631 | 0.105 | 0.369 | 0.777 |
| COPD |  | -0.081 | 0.370 | 0.827 | -0.253 | 1.115 | 0.821 | 2.549 | 3.324 | 0.443 | -3.073 | 22.432 | 0.891 | 0.337 | 0.720 | 0.640 |
| Peptic ulcer |  | -0.011 | 0.198 | 0.957 | -0.150 | 0.598 | 0.802 | 1.354 | 1.784 | 0.448 | -4.419 | 11.876 | 0.710 | -0.092 | 0.386 | 0.813 |
| Atrial fibrillation |  | 0.248 | 0.382 | 0.516 | 0.344 | 1.152 | 0.765 | -0.040 | 3.318 | 0.990 | -6.506 | 22.230 | 0.770 | -0.110 | 0.723 | 0.879 |
| Hypertension |  | -0.479 | 0.235 | 0.042* | -1.366 | 0.708 | 0.054 | -4.451 | 2.050 | 0.030* | 22.884 | 13.905 | 0.100 | 0.252 | 0.446 | 0.572 |
| CD |  | -0.127 | 0.301 | 0.672 | -0.838 | 0.905 | 0.354 | 2.539 | 2.695 | 0.346 | -19.26 | 18.217 | 0.290 | 0.270 | 0.584 | 0.643 |
| Ischemic heart disease |  | -0.104 | 0.249 | 0.675 | 0.334 | 0.753 | 0.657 | -3.405 | 2.171 | 0.117 | 13.315 | 14.753 | 0.367 | 0.488 | 0.474 | 0.303 |
| Ischemic stroke |  | -0.016 | 0.333 | 0.961 | -0.442 | 1.005 | 0.660 | 3.070 | 2.902 | 0.290 | 4.888 | 19.686 | 0.804 | 0.425 | 0.632 | 0.501 |
| Number of inpatient records |  | -0.021 | 0.009 | 0.023* | -0.026 | 0.028 | 0.353 | -0.140 | 0.034 | <.001* | 0.167 | 0.375 | 0.656 | 0.046 | 0.016 | 0.003* |
| time |  | 0.003 | 0.002 | 0.036* | 0.004 | 0.005 | 0.347 | 0.007 | 0.003 | 0.034* | -0.468 | 0.062 | <.001* | -0.001 | 0.003 | 0.621 |
| Diabetes and time |  | -0.004 | 0.001 | 0.003* |  |  |  | 0.015 | 0.004 | <.001* | -0.284 | 0.053 | <.001* | -0.015 | 0.002 | <.001* |
| Cerebrovascular accident and time |  | -0.007 | 0.002 | <.001* | -0.023 | 0.005 | <.001* | -0.019 | 0.006 | 0.002* | 0.247 | 0.069 | <.001* | 0.006 | 0.003 | 0.038* |
| Heart failure and time |  | -0.003 | 0.001 | 0.011* | -0.009 | 0.004 | 0.018* | -0.027 | 0.005 | <.001* |  |  |  |  |  |  |
| Heart arrhythmia and time |  |  |  |  | -0.010 | 0.005 | 0.032* | 0.012 | 0.006 | 0.038* | -0.210 | 0.066 | 0.001* | 0.005 | 0.003 | 0.118 |
| CAHD and time |  |  |  |  |  |  |  |  |  |  |  |  |  | 0.004 | 0.003 | 0.084 |
| Anemia and time |  |  |  |  |  |  |  | 0.017 | 0.004 | <.001* | -0.119 | 0.048 | 0.014* | 0.005 | 0.002 | 0.034* |
| Dyslipidemia and time |  |  |  |  |  |  |  |  |  |  | -0.110 | 0.063 | 0.080 |  |  |  |
| COPD and time |  |  |  |  |  |  |  | -0.036 | 0.010 | <.001* | 0.297 | 0.116 | 0.011* | 0.016 | 0.005 | 0.002* |
| Peptic ulcer and time |  |  |  |  |  |  |  | -0.021 | 0.005 | <.001* |  |  |  | 0.007 | 0.003 | 0.006* |
| Atrial fibrillation and time |  | -0.011 | 0.003 | <.001* | -0.025 | 0.009 | 0.006* |  |  |  |  |  |  | 0.011 | 0.005 | 0.026* |
| Hypertension and time |  | 0.011 | 0.002 | <.001* | 0.032 | 0.005 | <.001* |  |  |  | -0.099 | 0.074 | 0.185 | -0.006 | 0.003 | 0.044* |
| CD and time |  |  |  |  |  |  |  | 0.019 | 0.008 | 0.016* | 0.210 | 0.095 | 0.027* | 0.015 | 0.004 | <.001* |
| Ischemic heart disease and time |  | 0.009 | 0.002 | <.001* | 0.021 | 0.006 | <.001* |  |  |  | 0.095 | 0.079 | 0.230 | -0.005 | 0.003 | 0.129 |
| Ischemic stroke and time |  | 0.013 | 0.002 | <.001* | 0.043 | 0.008 | <.001* |  |  |  | 0.173 | 0.102 | 0.090 | -0.009 | 0.004 | 0.046* |
| UN(1,1) |  | 0.646 | 0.082 | <.001* | 5.865 | 0.746 | <.001* | 52.466 | 6.593 | <.001* | 2343.7 | 294.63 | <.001* | 2.349 | 0.297 | <.001* |
| Residual |  | 0.809 | 0.012 | <.001* | 7.586 | 0.116 | <.001* | 10.055 | 0.156 | <.001* | 1328.5 | 20.280 | <.001* | 2.359 | 0.036 | <.001* |

* p≤0.05

Appendix 2. Estimation results of time-based explanatory variables in the random intercept model for a biochemical test (ALT, AST, albumin, alkaline, protein, total bilirubin levels)

| Variable Categorical | | Outcomes | | | | | | | | | | | | | | | | | |
| --- | --- | --- | --- | --- | --- | --- | --- | --- | --- | --- | --- | --- | --- | --- | --- | --- | --- | --- | --- |
|  |  | ALT | | | AST | | | Albumin | | | Alkaline | | | Protein | | | Total Bilrubin | | |
|  |  | Estimate | SE | P | Estimate | SE | P | Estimate | SE | P | Estimate | SE | P | Estimate | SE | P | Estimate | SE | P |
| Intercept |  | 25.035 | 3.632 | <.001* | 21.605 | 3.163 | <.001* | 4.195 | 0.123 | <.001* | 91.722 | 12.116 | <.001* | 7.345 | 0.205 | <.001* | 0.428 | 0.063 | <.001* |
| Male |  | 0.165 | 1.388 | 0.906 | -1.382 | 1.210 | 0.254 | 0.021 | 0.047 | 0.650 | -6.404 | 4.608 | 0.165 | -0.091 | 0.078 | 0.244 | 0.048 | 0.024 | 0.041* |
| Age |  | -0.056 | 0.053 | 0.297 | 0.048 | 0.047 | 0.300 | -0.003 | 0.002 | 0.139 | 0.131 | 0.177 | 0.459 | -0.001 | 0.003 | 0.673 | -0.000 | 0.001 | 0.600 |
| Duration of hemodialysis treatment | Less than 1 year | -1.846 | 1.685 | 0.273 | -2.310 | 1.470 | 0.116 | -0.064 | 0.057 | 0.262 | -7.104 | 5.575 | 0.203 | -0.133 | 0.095 | 0.161 | -0.021 | 0.029 | 0.455 |
|  | More than 1years | -1.551 | 1.596 | 0.331 | 0.237 | 1.392 | 0.865 | 0.067 | 0.054 | 0.220 | -1.063 | 5.247 | 0.839 | -0.220 | 0.089 | 0.014* | -0.009 | 0.027 | 0.748 |
| Diabetes |  | -0.712 | 1.570 | 0.650 | -1.725 | 1.369 | 0.208 | -0.071 | 0.055 | 0.198 | 1.434 | 5.217 | 0.783 | -0.042 | 0.091 | 0.649 | -0.014 | 0.027 | 0.600 |
| Cerebrovascular accident |  | -1.970 | 2.227 | 0.376 | -2.297 | 1.779 | 0.197 | 0.017 | 0.071 | 0.808 | -4.541 | 6.986 | 0.516 | 0.067 | 0.114 | 0.560 | -0.017 | 0.035 | 0.627 |
| Heart failure |  | -1.837 | 1.722 | 0.286 | -0.442 | 1.383 | 0.749 | -0.007 | 0.054 | 0.897 | -1.192 | 5.395 | 0.825 | -0.021 | 0.091 | 0.819 | 0.046 | 0.027 | 0.089 |
| Heart arrhythmia |  | 1.371 | 2.269 | 0.546 | 0.892 | 1.835 | 0.627 | 0.022 | 0.074 | 0.768 | -5.212 | 6.903 | 0.450 | 0.126 | 0.121 | 0.298 | -0.021 | 0.036 | 0.565 |
| CAHD |  | -1.074 | 1.730 | 0.535 | -0.229 | 1.509 | 0.879 | 0.049 | 0.061 | 0.421 | 2.859 | 5.716 | 0.617 | -0.023 | 0.097 | 0.812 | 0.018 | 0.037 | 0.615 |
| Anemia |  | -2.926 | 1.593 | 0.066 | -0.607 | 1.278 | 0.635 | -0.038 | 0.050 | 0.446 | 6.440 | 4.852 | 0.184 | -0.190 | 0.085 | 0.026* | -0.064 | 0.032 | 0.043* |
| Dyslipidemia |  | -2.063 | 1.985 | 0.299 | 1.373 | 1.591 | 0.388 | 0.050 | 0.062 | 0.418 | 10.138 | 6.231 | 0.104 | 0.102 | 0.105 | 0.332 | 0.018 | 0.031 | 0.560 |
| COPD |  | -3.043 | 3.478 | 0.382 | -2.637 | 3.032 | 0.384 | -0.349 | 0.122 | 0.004* | -12.80 | 11.866 | 0.281 | -0.577 | 0.200 | 0.004* | -0.032 | 0.059 | 0.583 |
| Peptic ulcer |  | 0.905 | 2.034 | 0.656 | 3.060 | 1.626 | 0.060 | -0.095 | 0.063 | 0.134 | 13.214 | 6.379 | 0.038* | 0.160 | 0.108 | 0.137 | 0.050 | 0.032 | 0.115 |
| Atrial fibrillation |  | 0.385 | 3.492 | 0.912 | 0.386 | 3.047 | 0.899 | 0.055 | 0.122 | 0.651 | -18.68 | 11.434 | 0.102 | -0.164 | 0.195 | 0.400 | -0.031 | 0.078 | 0.689 |
| Hypertension |  | 2.886 | 2.158 | 0.181 | 0.364 | 1.884 | 0.847 | -0.054 | 0.075 | 0.478 | -12.70 | 7.374 | 0.085 | -0.080 | 0.125 | 0.521 | -0.005 | 0.037 | 0.892 |
| CD |  | 5.155 | 3.073 | 0.093 | 2.665 | 2.697 | 0.323 | -0.025 | 0.099 | 0.801 | -7.924 | 9.579 | 0.408 | -0.261 | 0.162 | 0.108 | -0.039 | 0.061 | 0.518 |
| Ischemic heart disease |  | 3.535 | 2.510 | 0.159 | 1.867 | 1.994 | 0.349 | -0.049 | 0.080 | 0.542 | 5.641 | 7.792 | 0.469 | 0.267 | 0.132 | 0.043* | 0.022 | 0.052 | 0.662 |
| Ischemic stroke |  | 1.668 | 3.331 | 0.617 | 0.184 | 2.997 | 0.951 | 0.128 | 0.104 | 0.219 | -2.113 | 10.398 | 0.839 | 0.163 | 0.175 | 0.353 | 0.014 | 0.052 | 0.784 |
| Number of inpatient records |  | -0.264 | 0.164 | 0.106 | 0.038 | 0.178 | 0.832 | -0.024 | 0.003 | <.001* | 1.087 | 0.349 | 0.002* | -0.011 | 0.005 | 0.031* | -0.002 | 0.005 | 0.666 |
| time |  | -0.050 | 0.016 | 0.001* | 0.008 | 0.011 | 0.478 | -0.001 | 0.001 | 0.217 | -0.201 | 0.058 | <.001* | -0.009 | 0.001 | <.001* | 0.000 | 0.001 | 0.991 |
| Diabetes and time |  |  |  |  |  |  |  | -0.001 | 0.000 | 0.001* |  |  |  | 0.004 | 0.001 | <.001* |  |  |  |
| Cerebrovascular accident and time |  | 0.070 | 0.030 | 0.018* |  |  |  | -0.003 | 0.001 | <.001* | 0.380 | 0.063 | <.001* |  |  |  |  |  |  |
| Heart failure and time |  | 0.059 | 0.022 | 0.008* |  |  |  |  |  |  | 0.201 | 0.048 | <.001* | 0.001 | 0.001 | 0.067 |  |  |  |
| Heart arrhythmia and time |  | -0.037 | 0.028 | 0.193 |  |  |  | 0.002 | 0.001 | 0.006* |  |  |  | -0.007 | 0.001 | <.001* |  |  |  |
| CAHD and time |  |  |  |  |  |  |  | -0.002 | 0.000 | 0.002* |  |  |  |  |  |  | 0.001 | 0.001 | 0.486 |
| Anemia and time |  | 0.044 | 0.021 | 0.033* |  |  |  |  |  |  |  |  |  | 0.006 | 0.001 | <.001* | 0.001 | 0.001 | 0.089 |
| Dyslipidemia and time |  | 0.047 | 0.026 | 0.070 |  |  |  |  |  |  | -0.102 | 0.056 | 0.070 | -0.003 | 0.001 | <.001* |  |  |  |
| COPD and time |  |  |  |  |  |  |  | 0.007 | 0.001 | <.001* | 0.346 | 0.108 | 0.001* | 0.007 | 0.002 | <.001* |  |  |  |
| Peptic ulcer and time |  | -0.055 | 0.027 | 0.041* |  |  |  |  |  |  | -0.101 | 0.057 | 0.077 | -0.006 | 0.001 | <.001* |  |  |  |
| Atrial fibrillation and time |  |  |  |  |  |  |  | -0.001 | 0.001 | 0.144 |  |  |  |  |  |  | 0.002 | 0.002 | 0.308 |
| Hypertension and time |  |  |  |  |  |  |  | 0.003 | 0.001 | <.001* | 0.273 | 0.066 | <.001* | 0.007 | 0.001 | <.001* |  |  |  |
| CD and time |  | -0.098 | 0.040 | 0.015* | -0.063 | 0.036 | 0.082 | -0.002 | 0.001 | 0.005* | -0.133 | 0.083 | 0.109 | 0.005 | 0.001 | <.001* | 0.002 | 0.001 | 0.054 |
| Ischemic heart disease and time |  | -0.049 | 0.034 | 0.148 |  |  |  | 0.002 | 0.001 | 0.004* | 0.192 | 0.071 | 0.007* | -0.003 | 0.001 | 0.020* | -0.002 | 0.001 | 0.108 |
| Ischemic stroke and time |  | 0.094 | 0.045 | 0.035* | 0.065 | 0.046 | 0.159 |  |  |  | 0.195 | 0.096 | 0.043* | -0.007 | 0.001 | <.001* |  |  |  |
| UN(1,1) |  | 54.130 | 7.264 | <.001* | 39.327 | 5.527 | <.001* | 0.066 | 0.008 | <.001* | 605.14 | 78.708 | <.001* | 0.177 | 0.023 | <.001* | 0.012 | 0.002 | <.001* |
| Residual |  | 247.49 | 3.778 | <.001* | 296.56 | 4.524 | <.001* | 0.092 | 0.001 | <.001* | 1118.7 | 17.403 | <.001* | 0.244 | 0.004 | <.001* | 0.271 | 0.004 | <.001* |

* p≤0.05

Appendix 3. Estimation results of time-based explanatory variables in the random intercept model for a biochemical test(Creatinine, K, Na, Uric acid, Ca)

| Variable Categorical | | Outcomes | | | | | | | | | | | | | | |
| --- | --- | --- | --- | --- | --- | --- | --- | --- | --- | --- | --- | --- | --- | --- | --- | --- |
|  |  | Creatinine | | | K | | | Na | | | Uric | | | Ca | | |
|  |  | Estimate | SE | P | Estimate | SE | P | Estimate | SE | P | Estimate | SE | P | Estimate | SE | P |
| Intercept |  | 13.995 | 0.830 | <.001* | 4.344 | 0.217 | <.001* | 137.23 | 0.968 | <.001* | 7.892 | 0.514 | <.001* | 9.446 | 0.230 | <.001* |
| Male |  | 1.555 | 0.319 | <.001* | 0.021 | 0.084 | 0.802 | 0.140 | 0.368 | 0.704 | -0.010 | 0.195 | 0.961 | -0.210 | 0.088 | 0.016* |
| Age |  | -0.065 | 0.012 | <.001* | 0.000 | 0.003 | 0.964 | 0.013 | 0.014 | 0.352 | -0.027 | 0.007 | <.001* | 0.001 | 0.003 | 0.824 |
| Duration of hemodialysis treatment | Less than 1 year | 0.167 | 0.388 | 0.667 | 0.184 | 0.102 | 0.071 | 0.834 | 0.445 | 0.061 | 0.214 | 0.235 | 0.364 | -0.166 | 0.106 | 0.118 |
|  | More than 1years | 0.209 | 0.367 | 0.569 | 0.264 | 0.096 | 0.006* | 0.169 | 0.419 | 0.686 | 0.166 | 0.222 | 0.454 | 0.334 | 0.101 | <.001* |
| Diabetes |  | -1.287 | 0.367 | <.001* | -0.162 | 0.095 | 0.088 | -1.573 | 0.433 | <.001* | -0.091 | 0.219 | 0.679 | -0.281 | 0.099 | 0.005* |
| Cerebrovascular accident |  | 0.047 | 0.476 | 0.921 | -0.062 | 0.123 | 0.614 | 0.685 | 0.558 | 0.220 | 0.158 | 0.284 | 0.578 | -0.012 | 0.129 | 0.923 |
| Heart failure |  | -0.242 | 0.370 | 0.514 | -0.013 | 0.096 | 0.890 | 0.255 | 0.415 | 0.539 | 0.333 | 0.220 | 0.129 | -0.113 | 0.104 | 0.276 |
| Heart arrhythmia |  | 0.216 | 0.491 | 0.659 | -0.114 | 0.131 | 0.384 | -0.222 | 0.551 | 0.687 | -0.338 | 0.304 | 0.266 | 0.129 | 0.137 | 0.347 |
| CAHD |  | 0.483 | 0.404 | 0.232 | -0.053 | 0.108 | 0.623 | 0.183 | 0.471 | 0.698 | 0.355 | 0.240 | 0.140 | 0.141 | 0.109 | 0.198 |
| Anemia |  | -0.019 | 0.342 | 0.956 | 0.120 | 0.088 | 0.173 | -0.639 | 0.404 | 0.114 | -0.122 | 0.205 | 0.553 | -0.000 | 0.092 | 0.999 |
| Dyslipidemia |  | -0.046 | 0.426 | 0.914 | -0.007 | 0.113 | 0.952 | 0.511 | 0.497 | 0.304 | -0.086 | 0.252 | 0.732 | -0.094 | 0.119 | 0.430 |
| COPD |  | -0.413 | 0.801 | 0.606 | -0.136 | 0.217 | 0.529 | -0.256 | 0.911 | 0.779 | 0.289 | 0.481 | 0.548 | -0.319 | 0.229 | 0.163 |
| Peptic ulcer |  | 0.147 | 0.435 | 0.736 | -0.230 | 0.112 | 0.041* | 0.844 | 0.509 | 0.097 | -0.153 | 0.259 | 0.553 | 0.084 | 0.122 | 0.491 |
| Atrial fibrillation |  | 1.096 | 0.816 | 0.179 | 0.110 | 0.210 | 0.600 | -0.473 | 0.951 | 0.618 | 0.785 | 0.510 | 0.124 | 0.399 | 0.220 | 0.070 |
| Hypertension |  | 0.179 | 0.496 | 0.718 | 0.089 | 0.130 | 0.496 | 0.591 | 0.592 | 0.318 | 0.503 | 0.317 | 0.112 | 0.165 | 0.141 | 0.243 |
| CD |  | -0.157 | 0.650 | 0.809 | 0.356 | 0.176 | 0.043* | 0.381 | 0.738 | 0.606 | 0.112 | 0.407 | 0.783 | 0.121 | 0.178 | 0.499 |
| Ischemic heart disease |  | 0.512 | 0.526 | 0.330 | -0.189 | 0.143 | 0.185 | -0.190 | 0.597 | 0.750 | -0.291 | 0.316 | 0.358 | -0.152 | 0.150 | 0.313 |
| Ischemic stroke |  | 0.561 | 0.713 | 0.431 | 0.184 | 0.190 | 0.331 | 0.007 | 0.797 | 0.993 | 0.917 | 0.443 | 0.038* | -0.171 | 0.193 | 0.375 |
| Number of inpatient records |  | -0.045 | 0.014 | 0.002* | 0.010 | 0.006 | 0.080 | 0.012 | 0.028 | 0.657 | 0.068 | 0.019 | <.001* | -0.045 | 0.007 | <.001* |
| time |  | 0.008 | 0.001 | <.001* | 0.003 | 0.000 | <.001* | 0.015 | 0.005 | 0.002* | 0.009 | 0.003 | 0.001* | 0.003 | 0.001 | 0.007* |
| Diabetes and time |  | 0.019 | 0.002 | <.001* |  |  |  | 0.013 | 0.004 | 0.001* |  |  |  |  |  |  |
| Cerebrovascular accident and time |  | 0.003 | 0.003 | 0.284 |  |  |  | -0.006 | 0.005 | 0.251 |  |  |  |  |  |  |
| Heart failure and time |  | -0.005 | 0.002 | 0.018* |  |  |  |  |  |  |  |  |  | 0.001 | 0.001 | 0.154 |
| Heart arrhythmia and time |  | -0.016 | 0.003 | <.001* | 0.002 | 0.001 | 0.087 |  |  |  | -0.005 | 0.003 | 0.086 | -0.004 | 0.001 | 0.001* |
| CAHD and time |  | -0.006 | 0.002 | 0.013* | -0.002 | 0.001 | 0.078 | -0.005 | 0.004 | 0.169 |  |  |  |  |  |  |
| Anemia and time |  | 0.003 | 0.002 | 0.095 |  |  |  | 0.007 | 0.004 | 0.053 |  |  |  |  |  |  |
| Dyslipidemia and time |  | -0.004 | 0.002 | 0.101 | -0.002 | 0.001 | 0.009* | -0.009 | 0.005 | 0.038* |  |  |  | 0.002 | 0.001 | 0.024* |
| COPD and time |  |  |  |  | 0.004 | 0.002 | 0.022* |  |  |  |  |  |  | 0.010 | 0.002 | <.001* |
| Peptic ulcer and time |  | -0.017 | 0.002 | <.001* |  |  |  | -0.024 | 0.005 | <.001* |  |  |  | -0.002 | 0.001 | 0.046* |
| Atrial fibrillation and time |  | -0.014 | 0.005 | 0.002* |  |  |  | 0.027 | 0.009 | 0.002* | -0.008 | 0.005 | 0.132 |  |  |  |
| Hypertension and time |  |  |  |  |  |  |  | -0.017 | 0.006 | 0.002* | -0.009 | 0.003 | 0.005* | -0.003 | 0.001 | 0.041* |
| CD and time |  |  |  |  | -0.005 | 0.001 | <.001* |  |  |  | 0.010 | 0.004 | 0.012* |  |  |  |
| Ischemic heart disease and time |  |  |  |  | 0.004 | 0.001 | <.001* |  |  |  |  |  |  | 0.004 | 0.001 | 0.009* |
| Ischemic stroke and time |  | -0.016 | 0.004 | <.001* | -0.004 | 0.002 | 0.020* |  |  |  | -0.013 | 0.005 | 0.005* |  |  |  |
| UN(1,1) |  | 3.059 | 0.385 | <.001* | 0.207 | 0.026 | <.001* | 3.849 | 0.499 | <.001* | 1.065 | 0.141 | <.001* | 0.225 | 0.029 | <.001* |
| Residual |  | 1.939 | 0.030 | <.001* | 0.314 | 0.005 | <.001* | 7.397 | 0.115 | <.001* | 0.895 | 0.025 | <.001* | 0.442 | 0.007 | <.001* |

* p≤0.05
